# Gene expression studies of developing bovine *longissimus *muscle from two different beef cattle breeds

**DOI:** 10.1186/1471-213X-7-95

**Published:** 2007-08-16

**Authors:** Sigrid A Lehnert, Antonio Reverter, Keren A Byrne, Yonghong Wang, Greg S Nattrass, Nicholas J Hudson, Paul L Greenwood

**Affiliations:** 1 Cooperative Research Centre for Cattle and Beef Quality, Australia; 2 CSIRO Livestock Industries, Queensland Bioscience Precinct, 306 Carmody Road, St Lucia 4067, Australia; 3 South Australian Research & Development Institute (SARDI), Livestock Systems, Roseworthy 5371, Australia; 4 School of Integrative Biology, University of Queensland, St Lucia 4072, Australia; 5 Beef Industry Centre of Excellence, NSW Department of Primary Industries, JSF Barker Building, University of New England, Armidale 2351, Australia

## Abstract

**Background:**

The muscle fiber number and fiber composition of muscle is largely determined during prenatal development. In order to discover genes that are involved in determining adult muscle phenotypes, we studied the gene expression profile of developing fetal bovine *longissimus *muscle from animals with two different genetic backgrounds using a bovine cDNA microarray. Fetal *longissimus *muscle was sampled at 4 stages of myogenesis and muscle maturation: primary myogenesis (d 60), secondary myogenesis (d 135), as well as beginning (d 195) and final stages (birth) of functional differentiation of muscle fibers. All fetuses and newborns (total n = 24) were from Hereford dams and crossed with either Wagyu (high intramuscular fat) or Piedmontese (*GDF8 *mutant) sires, genotypes that vary markedly in muscle and compositional characteristics later in postnatal life.

**Results:**

We obtained expression profiles of three individuals for each time point and genotype to allow comparisons across time and between sire breeds. Quantitative reverse transcription-PCR analysis of RNA from developing *longissimus *muscle was able to validate the differential expression patterns observed for a selection of differentially expressed genes, with one exception. We detected large-scale changes in temporal gene expression between the four developmental stages in genes coding for extracellular matrix and for muscle fiber structural and metabolic proteins. *FSTL1 *and *IGFBP5 *were two genes implicated in growth and differentiation that showed developmentally regulated expression levels in fetal muscle. An abundantly expressed gene with no functional annotation was found to be developmentally regulated in the same manner as muscle structural proteins. We also observed differences in gene expression profiles between the two different sire breeds. Wagyu-sired calves showed higher expression of fatty acid binding protein 5 (*FABP5*) RNA at birth. The developing *longissimus *muscle of fetuses carrying the Piedmontese mutation shows an emphasis on glycolytic muscle biochemistry and a large-scale up-regulation of the translational machinery at birth. We also document evidence for timing differences in differentiation events between the two breeds.

**Conclusion:**

Taken together, these findings provide a detailed description of molecular events accompanying skeletal muscle differentiation in the bovine, as well as gene expression differences that may underpin the phenotype differences between the two breeds. In addition, this study has highlighted a non-coding RNA, which is abundantly expressed and developmentally regulated in bovine fetal muscle.

## Background

Genetic background and development prior to birth are known to influence the composition of bovine muscle tissue [1,2]. Prenatal muscle development is therefore a promising source of gene discovery for the molecular events that determine adult muscle phenotype.

Prenatal muscle development in mammals begins with the determination of myogenic cells (myoblasts) in the dermamyotome and their migration to their sites of differentiation [3]. Myoblasts then fuse to form multinucleated muscle cells, myotubes. In cattle, primary myotubes appear prior to 47 days of gestation [4]. Primary skeletal muscle fibers subsequently differentiate from this generation of myotubes [5]. Secondary muscle fibers are derived from a separate population of myotubes arising around day 90 [6].

The number of muscle fibers is roughly fixed at two thirds of bovine fetal development and muscle fibers differentiate before birth, making cattle a relatively mature species at birth compared to other mammals. During the final third of gestation in cattle, fetal isoforms of the muscle contractile proteins disappear and are replaced by adult isoforms. Also over the final third of gestation, metabolic differentiation takes place, with an increase in glycolytic and oxidative pathway enzymes [3]. Histologically, a steady decrease in the extracellular matrix contribution to muscle mass can be observed over fetal development in the bovine [7].

Large mammals such as pigs and cattle offer an advantageous model for the study of myogenesis *in vivo*, as individual muscles can be identified and reliably dissected from early stages. In addition, livestock breeds represent useful genetic models for muscle hypertrophy and other muscle traits, as these have been subject to intensive genetic selection due to their impact on meat quality. For example, array studies of pig fetal development have characterized the coordinate genome-wide expression changes during a developmental time course [8] and also exploited the contrasting muscle phenotypes of the Pietrain and Duroc pig breeds to compare prenatal events that may underlie the adult muscle phenotype [9].

Similarly, Sudre et al. [10] studied expression profiles of bovine fetal muscles using a human skeletal muscle macroarray. A number of human macroarray elements detected significant differential expression signals between fetal developmental stages. Their study highlighted the crucial developmental changes that occur during the final trimester of gestation, as most of the differentially expressed genes in this study were identified in the comparison between 210 and 260 d of gestation. A human microarray was employed in a study of fetal muscle from fetuses carrying myostatin loss-of-function mutations [11]. This work described breed-related differences in the expression of genes relevant to several skeletal muscle compartments (extracellular matrix, contractile cells and adipocytes).

In the present study, we used a bovine cDNA microarray derived from adult muscle and adipose tissue libraries to characterize genome-wide transcriptional changes that accompany muscle differentiation in cattle and to determine the impact of two contrasting genotypes on prenatal muscle development. We carried out a transcriptional profiling study of *longissimus *muscle from fetuses of both Wagyu × Hereford and Piedmontese × Hereford crosses, sampled at d 60, 135 and 195, as well as newborn calves. The double-muscled mutations of *GDF8 *carried by beef breeds such as Belgian Blue or Piedmontese cattle [1] have a large impact on muscle yield, and animals of the Japanese Black (or Wagyu) breed of cattle are genetically predisposed to accumulate intramuscular fat [12]. The postnatal phenotypes of offspring from Hereford dams sired by these breeds have been well-characterised and are highly divergent for muscle growth and intramuscular fat content [13]. The sampling time points chosen in this study represent primary myogenesis (d 60), secondary myogenesis (d 135), as well as beginning (d 195) and final stages (birth) of functional differentiation of muscle fibers.

## Results

The entire set of expression data was deposited on Gene Expression Omnibus database [14] and can be accessed using accession numbers GSM132170 and GSM132194. In this microarray profiling experiment of developing bovine *longissimus *muscle, we detected 174 individual genes (617 array elements) that met the criteria for differential gene expression [see Additional file 1] either across developmental time or between the two sire breeds.

### Temporal changes in gene expression

**Figure 1:**
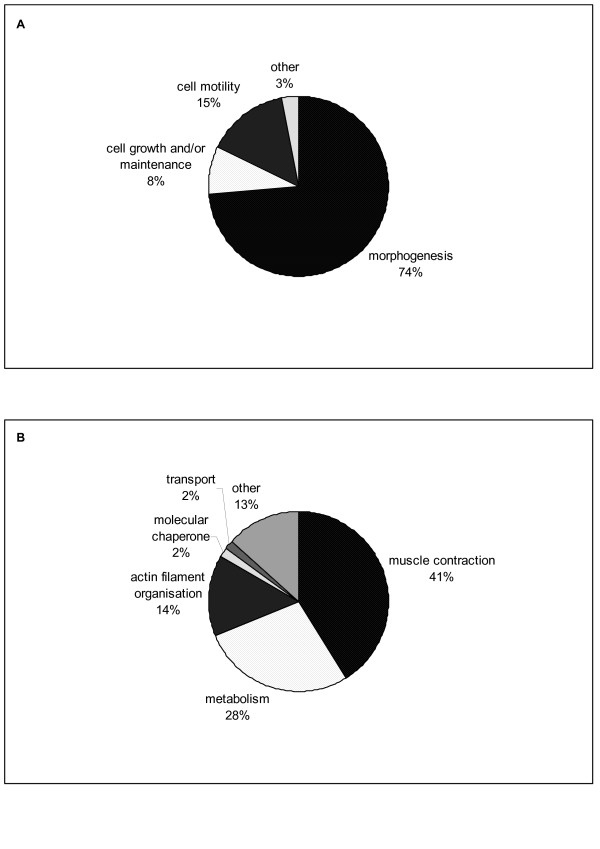
**Annotation of differentially expressed genes in bovine *longissimus *muscle**. The Expression Analysis Systematic Explorer (EASE) tool [41] was used to identify classes of overrepresented gene ontology annotations in the differentially expressed gene lists (Tables 1 and 2). (A) Biological process annotation of genes which showed decreasing expression over the developmental time course. (B) Biological process annotation of genes which showed increasing expression over the developmental time course.

Tables 1 and 2 list expression values for differentially expressed (DE) genes for which reliable annotations could be obtained using the Basic Local Alignment Search Tool (BLASTN [15]). These genes were differentially expressed across developmental time, regardless of breed. Twenty genes showed decreasing expression over time and 35 genes showed increasing gene expression over time. All genes in Tables 1 and 2 showed expression changes of more than 2-fold over the time course examined. Gene Ontology annotation of the DE genes using EASE [16] showed that genes involved with the biological processes, morphogenesis and cellular growth were overrepresented in the genes that show decreasing expression over time (Figure 1A). When annotations with respect to cellular component were examined, 69% of the genes with decreasing expression could be classified as belonging to "extracellular matrix" (data not shown). Collagen genes alone represent 68% of the DE genes in Table 1. On the other hand, in the list of genes with increasing expression over time, genes with biological process annotations belonging to muscle cell contraction and metabolism are overrepresented (Figure 1B). The main observation from the examination of the developmental time course is therefore that transcripts associated with extracellular matrix synthesis are increasingly less represented as a proportion of total tissue RNA as muscle development proceeds, while at the same time transcripts of muscle fiber structural, contractile and metabolism genes are gaining in prevalence when maturation of muscle fibres takes place in preparation for postnatal life. In most instances, the most marked increase or decrease in gene expression can be observed between d 195 of fetal development and birth (Tables 1 and 2).

**Table 1:** Genes showing decreasing expression in *longissimus *muscle of bovine fetuses over developmental time^1^

Gene^2^	GenBank Accession^3^	Number of array elements^4^	Gene expression ratio^5 ^135 d/60 d	Gene expression ratio^5 ^195 d/60 d	Gene expression ratio^5 ^birth/60 d
cadherin 11, type 2, OB-cadherin (osteoblast) (*CDH11*)	DW521798	1	0.26**	0.23**	0.10**
collagen, type I, alpha 1 (*COL1A1*)	CF613531	14	0.87	0.74	0.16**
collagen, type I, alpha 2 (*COL1A2*)	CF613950	9	0.75	0.64**	0.15**
collagen, type III, alpha 1 (*COL3A1*)	CF613798	29	0.72	0.78	0.15**
collagen, type XII, alpha 1 (*COL12A1*)	CF614200	1	0.48**	0.23**	0.12**
collagen, type XV, alpha 1(*COL15A1*)	DW521810	1	0.58**	0.41**	0.18**
fibrillin 1 (*FBN1*)	CF765332	4	0.57**	0.60**	0.23**
fibronectin 1 (*FN1*)	CF614211	7	0.39**	0.36**	0.08**
follistatin-like 1 (*FSTL1*)	CF613847	1	0.61**	0.35**	0.13**
guanine nucleotide binding protein (G protein), alpha inhibiting activity polypeptide 1 (*GNAI1*)	DW521797	1	0.58**	0.50**	0.20**
glypican 3 (*GPC3*)	DW521793	1	0.91	0.84	0.11**
Insulin-like growth factor binding protein 5 (*IGFBP5*)	CF614176	1	0.71	0.72	0.21**
lumican (*LUM*)	CF613654	2	0.79	0.64**	0.16**
osteoglycin (*OGN*)	CF614268	1	0.59**	0.67*	0.19**
plastin 3 (T isoform) (*PLS3*)	DW521782	1	0.49**	0.43**	0.20**
osteonectin (*SPARC*)	CF613734	8	0.69*	0.68*	0.21**
osteopontin (*SPP1*)	DW521777	3	0.53**	0.19**	0.15**
stathmin 1/oncoprotein 18 (*STMN1*)	DW521774	1	0.54**	0.38**	0.10**
tubulin, alpha 1 (*TUBA1*)	CF613655	2	0.54**	0.46**	0.17**
vimentin (*VIM*)	CF613639	7	0.51**	0.49**	0.16**

^1^* indicates a statistically significant decrease in gene expression at P < 0.05 level; ** indicates a statistically significant decrease in gene expression at P < 0.01 level.

^2^Sequence annotation was carried out using approved gene symbols (human genome nomenclature) *via *the IBISS4 database [17]. BLAST [15] scores > 110 and e-values < 2 E-23 were accepted for annotation purposes.

^3^in cases where more than 1 microarray element was used to calculate the expression data, the accession number of 1 representative element is shown.

^4^Number of microarray elements representing the same gene that were used to calculate the expression data.

^5^To obtain the ratios shown, averaged absolute microarray signal intensity values for d 135, 195 and birth were divided by the signal intensities measured at d 60.

**Table 2:** Genes showing increasing expression in *longissimus *muscle of bovine fetuses over developmental time^1^

Gene^2^	GenBank Accession^3^	Number of array elements^4^	Gene expression ratio^5 ^135 d/60 d	Gene expression ratio^5 ^195 d/60 d	Gene expression ratio^5 ^birth/60 d
actin, alpha 1, skeletal muscle (*ACTA1*)	CF614694	6	0.80	2.49**	4.09**
actinin, alpha 3 (*ACTN3*)	CF614924	3	0.62	3.79**	5.63**
adenylate kinase 1 (*AK1*)	CF614671	1	1.38**	4.25**	4.60**
aldolase A, fructose-biphosphate (*ALDOA*)	CF615006	1	1.28*	3.27**	5.82**
ankyrin repeat domain 1 (cardiac muscle) (*ANKRD1*)	CF614403	2	0.83	2.62**	6.62**
mitochondrially encoded ATP synthase 6 (*MT-ATP6*)	CF613499	1	1.10	2.69**	5.48**
chaperone, ABC1 activity of bc1 complex like (S. pombe) (*CABC1*)	CF615094	1	0.51	1.18	6.43**
calsequestrin 1 (fast-twitch, skeletal muscle) (*CASQ1*)	CO729180	1	1.08	3.93**	5.00**
creatine kinase, muscle (*CKM*)	CF614901	15	2.15**	3.04**	7.49**
creatine kinase, mitochondrial 2 (sarcomeric) (*CKMT2*)	CF614479	1	0.60	1.76**	10.08**
cardiomyopathy associated 5 (*CMYA5*)	CF615342	4	0.77	1.63**	4.79**
cold shock domain protein A (*CSDA*)	CF615315	3	0.64	1.74**	4.71**
cytochrome C (*CYC1*)	CF614629	1	2.14**	2.53**	6.90**
enolase 3 (beta, muscle) (*ENO3*)	CF614534	16	1.93**	3.58**	5.25**
fructose-1,6-bisphosphatase 2 (*FBP2*)	CF614569	2	0.53	2.39**	6.73**
heat shock 70 kDa protein 1B (*HSPA1B*)	CF614728	1	1.03	2.17**	4.02**
heat shock 90 kDa protein 1, alpha (*HSPCA*)	DW521445	1	1.30	3.63**	6.10**
lactate dehydrogenase A (*LDHA*)	CF614400	5	0.70	2.40**	4.31**
similar to dysferlin interacting protein 1, transcript variant 1 (LOC616223), mRNA	CO729195	2	1.57**	3.62**	4.97**
myoglobin (*MB*)	CF615014	18	1.79**	2.51**	6.68**
Myosin binding protein C, fast type (*MYBPC2*)	CF615254	14	0.89	2.73**	9.83**
myosin, heavy polypeptide 1, skeletal muscle, adult (*MYH1*)	CF615300	3	0.59	2.70**	5.85**
myosin, heavy polypeptide 2, skeletal muscle, adult (*MYH2*)	CF614936	13	1.40**	2.70**	5.37**
myozenin 1 (*MYOZ1*)	CF614412	18	2.22**	2.89**	7.66**
ncRNA orthologous with *NEAT1*	CF614858	12	0.91	1.97**	6.75**
PDZ and LIM domain 3 (*PDLIM3*)	CF615153	10	0.92	3.88**	5.05**
6-phosphofructo-2-kinase/fructose-2,6-biphosphatase 3 (*PFKFB3*)	CF615087	1	0.36	0.51	6.27**
phosphofructokinase, muscle (*PFKM*)	DW521807	1	1.01	4.12**	6.71**
phosphoglycerate mutase 2 (muscle) (*PGAM2*)	CF614969	6	1.12	4.03**	4.65**
phosphoglucomutase 1 (*PGM1*)	CF615240	7	1.06	3.50**	5.47**
Phosphorylase, glycogen; muscle (*PYGM*)	CF614749	6	1.06	3.17**	7.27**
solute carrier family 25 (mitochondrial carrier; adenine nucleotide translocator), member 4 (*SLC25A4*)	CF614535	3	0.80	1.93**	5.14**
titin-cap (telethonin) (*TCAP*)	CF614598	5	2.81**	4.15**	8.78**
tropomodulin 4 (muscle) (*TMOD4*)	CF614407	2	0.79	3.62**	6.53**
Titin immunoglobulin domain protein (myotitilin) (*TTID*)	CF614674	5	0.58	1.63**	4.61**

^1^* indicates a statistically significant increase in gene expression at P < 0.05 level; ** indicates a statistically significant increase in gene expression at P < 0.01 level.

^2^Sequence annotation was carried out using approved gene symbols (human genome nomenclature) *via *the IBISS4 database [17]. BLAST [15] scores > 110 and e-values < 2 E-23 were accepted for annotation purposes.

^3^In cases where more than 1 microarray element was used to calculate the expression data, the accession number of 1 representative element is shown.

^4^Number of microarray elements representing the same gene that were used to calculate the expression data.

^5^To obtain the ratios shown, averaged absolute microarray signal intensity values for d 135, 195 and birth were divided by the signal intensities measured at d 60.

Two genes, *FSTL1 *and *IGFBP5 *(Table 1), which are implicated in the regulation of cell growth and differentiation, are significantly downregulated in developing bovine muscle. These genes were chosen for quantitative reverse transcription-PCR (qRT-PCR) validation (see below).

Twelve microarray probes that reported increasing expression levels during development all matched to a single consensus sequence (btcn26022) in the IBISS4 database [17]. Using the NETBLAST function in IBISS4, a match with a non-coding (nc) RNA transcript orthologous with the recently described *NEAT1 *transcript was identified [18] (Table 2).

### Gene expression differences correlated with sire breed

A subset of gene expression differences according to sire breed is summarized in Table 3. In total, 82 genes were differentially expressed according to the breed of the fetus' sire in at least one time point [see Additional file 2]. The temporal changes in gene expression profiles were greater than the expression differences that were correlated to sire breed. Only 3 of these DE genes (*ALDOA*, *FABP5 *and *MATR3*) show a fold-change of more than 2 [see Additional file 2]. *ALDOA *was around two-fold less expressed in the *longissimus *muscle of Wagyu-sired fetuses at d195, while *FABP5 *showed more than two-fold less expression in the *longissimus *muscle of Piedmontese-sired calves at birth. *FABP4 *expression is also highlighted in Table 3, as this gene showed less gene expression in the *longissimus *muscle of Piedmontese-sired fetuses samples at d 135, but at no other time point. *MATR3 *showed less gene expression in the *longissimus *muscle of day 60 Wagyu-sired fetuses.

**Table 3:** Examples of genes showing differential expression in *longissimus *muscle of bovine fetuses from different sire breeds in at least one time point^1^

Gene^2^	GenBank Accession^3^	Number of array elements^4^	Gene expression ratio^5 ^P60d/W60d	Gene expression ratio^5 ^P135d/W135d	Gene expression ratio^5 ^P195d/W195d	Gene expression ratio^5 ^Pbirth/Wbirth
Aldolase A, fructose-biphosphate (*ALDOA*)	CF615006	4	0.87	1.04	2.16**	0.90
Collagens (*COL1A1*, *COL1A2*, *COL3A1*, *COL12A1*, *COL15A1*)	Additional file 2	42	1.21	0.70	0.96	0.64**
Cytochrome c oxidases (*COX5B*, *6C*, *7B*, *7C*)	Additional file 2	9	0.93	0.99	0.84	1.74**
Fatty acid binding protein 4, adipocyte (*FABP4*)	CF614083	3	1.01	0.70**	0.90	0.98
Fatty acid binding protein 5 (*FABP5*)	CF613827	1	1.29	0.93	1.16	0.44**
Matrin 3 (*MATR3*)	CF614914	1	2.35**	1.15	0.79	0.64**
Ribosomal L proteins (*RPL*)	Additional file 2	32	0.75	0.93	1.01	1.52**
Ribosomal S proteins (*RPS*)	Additional file 2	13	0.80	0.89	0.99	1.51**

^1^* indicates a statistically significant gene expression difference at P < 0.05 level; ** indicates a statistically significant gene expression difference at P < 0.01 level.

^2^Sequence annotation was carried out using approved gene symbols (human genome nomenclature) *via *the IBISS4 database [17]. BLAST [15] scores > 110 and e-values < 2 E-23 were accepted for annotation purposes.

^3^in cases where more than 1 microarray element was used to calculate the expression data, the accession number of 1 representative element is shown.

^4^Number of microarray elements representing the same gene that were used to calculate the expression data.

^5^To obtain the ratios shown, averaged absolute microarray signal intensity values for P (Piedmontese × Hereford) fetuses were divided by the signal intensities measured for W (Wagyu × Hereford) fetuses at d 60, d 135, d 195 and birth.

A number of genes showed smaller, yet still significant differences in gene expression between breeds in at least one time point [see Additional file 2]. Table 3 provides a summary for some of these classes of genes, for example the collagen genes, which were more highly represented in the transcriptome of late-stage Wagyu fetuses. All differentially expressed ribosomal protein genes and cytochrome c oxidase genes were detected as significantly up-regulated in the muscle of Piedmontese crossbred calves at birth (Table 3 and [Additional file 2]). Ribosomal protein and cytochrome c oxidase genes were not detected as differentially expressed over developmental time (Tables 1 and 2).

### qRT-PCR validation of microarray gene expression patterns

We conducted qRT-PCR studies on the amplified RNA used in the microarray study. Assays for *FABP4*, *FABP5*, *FSTL1*, *IGFBP5*, and *MATR3 *were developed to confirm temporal and sire-breed specific gene expression differences. In order to confirm that breed-specific differences caused by the Piedmontese myostatin (*GDF8*) mutation could be detected by gene expression measurements, and because *GDF8 *was not represented on the microarray, *GDF8 *expression was studied using qRT-PCR. Goodness of fit, as measured by the R^2^, resulted in the ANOVA model explaining 88.7%, 80.7%, 63.6%, 87.7%, 94.1% and 98.3% of the total variation in the expression of *GDF8*, *FABP4*, *FABP5*, *IGFBP5*, *FSTL1*, and *MATR3*, respectively [see Additional file 3]. Temporal gene expression patterns detected by microarray were confirmed for *IGFBP5 *and *FSTL1 *(Table 4). The breed-specific differences in *FABP4 *and *FABP5 *expression documented by microarray were accurately confirmed by qRT-PCR (Table 5). *FABP4 *expression was higher in the *longissimus *muscle of Wagyu-sired fetuses at d195 and birth. *FABP5 *expression was higher in the *longissimus *muscle of Wagyu-sired calves at birth, but no significant differences were detected at any other time point. *GDF8 *expression was significantly higher in the *longissimus *muscle of Piedmontese-sired fetuses at d60, d135 and birth (Table 5). No difference could be detected at d195.

**Table 4:** Quantitative reverse transcription PCR (qRT-PCR) measurements of gene expression in total RNA from *longissimus *muscle of bovine fetuses – gene expression ratios over developmental time

Gene	Fold Ratio^1^		
	135 d/60 d	195 d/60 d	birth/60 d

IGF-binding protein 5 (*IGFBP5*)	0.97	0.55 **	0.08 **
Follistatin-like (*FSTL1*)	0.62 **	0.24 **	0.05 **

^1^To obtain fold ratios, mean normalized (from ANOVA and against 18S RNA) qRT-PCR values at 135 d gestation, 195 d gestation and birth were divided by the values at 60 d gestation.

** Significance at P < 0.01.

**Table 5:** Quantitative reverse transcription PCR (qRT-PCR) measurements of gene expression in total RNA from *longissimus *muscle of bovine fetuses – gene expression ratios between breeds

Gene	Fold Ratio^1^			
	P60d/W60d	P135d/W135d	P195d/W195d	Pbirth/Wbirth

Myostatin (*GDF8*)	1.52 **	1.45 *	1.15	1.51 **
Fatty-acid binding protein 4 (*FABP4*)	1.32	0.56	0.14 **	0.20 **
Fatty-acid binding protein 5 (*FABP5*)	0.88	1.09	1.00	0.54 **
Matrin 3 (*MATR3*)	1.10	1.10	0.96	0.75

^1^To obtain fold ratios, mean normalized (from ANOVA and against 18S RNA) qRT-PCR values from P (Piedmontese × Hereford) samples were divided by those from W (Wagyu × Hereford) samples at 60 d gestation, 135 d gestation, 195 d gestation and birth.

* Significance at P < 0.05; ** Significance at P < 0.01.

**Figure 2:**
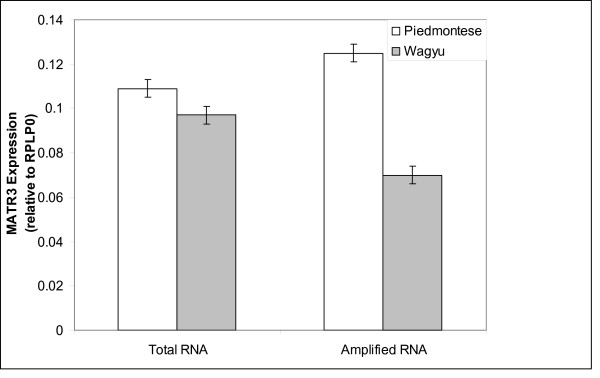
**Quantitative reverse transcription (qRT-PCR) measurements of matrin 3 (*MATR3*) gene expression in bovine d 60 *longissimus *muscle**. qRT-PCR was used to measure *MATR3 *expression from Hereford × Piedmontese (clear) or Hereford × Wagyu (shaded) fetuses, comparing total and amplified RNA. The data represent average values and standard error measurements from 3 individual animal samples, normalized against ribosomal protein, large PO (*RPLPO*) expression measured in the same sample.

Using the *MATR3 *qRT-PCR assay, we were unable to confirm the >2 fold higher gene expression detected by microarray in d60 muscle RNA samples from Wagyu-sired fetuses (Table 5). In order to clarify what may have caused this discrepancy, qRT-PCR assays for *MATR3 *were performed on the amplified RNA samples that had been used for the microarray analysis. This analysis showed that the RNA samples amplified from the d 60 *longissimus *muscle of Piedmontese-sired fetuses contained higher amounts of *MATR3 *RNA than the non-amplified RNA sample (Figure 2). Specifically, this elevated expression was due to one individual RNA sample (P663, data not shown). qRT-PCR testing of *MATR3 *expression therefore confirmed that the microarray analysis, which was carried out with amplified RNA, had accurately identified the elevated *MATR3 *expression levels in the d 60 sample. The results did, however uncover a discrepancy between amplified and total RNA for at least one particular individual and one gene.

## Discussion

This study was designed to map out gene expression changes that accompany four discrete phases of muscle development: the first wave of myogenesis (d 60), the second generation myoblast formation (d 135) and the beginning (d 195) and end (birth) stages of contractile and metabolic differentiation of muscle fibers. As two different breeds of sire (Wagyu and Piedmontese) were used to generate the fetuses in this study, we also attempted to detect gene expression differences that may be associated with the genetic background of the fetus. The fact that Hereford crossbreeds rather than purebred fetuses were used in this comparison is a limitation imposed by resource constraints. However, the postnatal phenotypes of offspring from Hereford dams sired by these breeds have been studied in a separate investigation and were found to be highly divergent for muscle growth and intramuscular fat content [13].

At d 60, the *longissimus *muscle was not identifiable as a distinct anatomical entity. An area of tissue corresponding to the future position of the *longissimus *muscle was therefore collected at this time point. At the d 60 time point it was more problematic to accurately dissect the overlying skin tissue away from the muscle. Reliable and consistent sampling of the *longissimus *muscle, using position along the vertebral column as a positional marker, was achieved from d 135 onwards. Due to limiting sample amounts, particularly at the d 60 time point, all RNA samples were subjected to a linear amplification procedure before labelling for microarray hybridization.

The gene expression analysis of *longissimus *muscle RNA samples provided evidence that the linear amplification procedure may have introduced bias into the representation of mRNA sequences in the d 60 microarray sample from one individual. While numerous studies have shown that linear amplification will conserve transcript abundance to an acceptable level, the potential to introduce significant bias to gene expression studies does exist [19]. For the above reasons, the discussion of differential gene expression will focus on the time points d 135, d 195 and birth.

### Temporal changes in gene expression

This microarray study of fetal bovine muscle development detected a large number of differentially expressed genes across developmental time. The expression pattern of muscle fiber structural and metabolism genes observed in this study is correlated to the timing of contractile differentiation of muscle fibers during fetal development. The overall trend of decreasing representation of extracellular matrix gene expression in fetal bovine muscle over time is in accordance with histological observations that show the proportion of extracellular matrix material on a muscle section decreasing from 70% to 27% over the time course studied here [7]. The increasing representation of muscle fiber-specific gene expression over time may therefore be a reflection of the proliferation and differentiation of muscle fibers within a connective tissue "scaffold" established early during development [20].

An earlier transcriptome study of bovine muscles during ontogenesis [10] did not identify connective tissue gene expression as a significant contributor to differential gene expression across developmental time. This may be a consequence of the skeletal muscle focused macroarray that was used in their study. The bovine fat/muscle microarray used in this study was constructed from muscle and subcutaneous fat cDNA libraries [21]. The differentially expressed microarray elements that showed decreasing gene expression over time, mainly had their origin in the subcutaneous fat cDNA library.

The observation that the most marked gene expression changes occur between d 195 and birth is in accordance with other studies that have shown the dramatic adaptations of skeletal muscle in preparation for birth [10]. As the postnatal muscle samples studied here were collected within 24 hours after birth, our study may, in addition, detect gene expression differences that reflect birth trauma.

We were unable to detect transcriptional changes that reflect the replacement of fetal with adult isoforms of contractile protein transcripts during the final trimester of pregnancy, as we are using a microarray based on adult skeletal muscle gene expression [21].

The vast majority of temporal changes in gene expression in fetal *longissimus *muscle comprise structural and metabolic components of extracellular matrix and muscle fibers. Two exceptions are *FSTL1*and *IGFBP5*, which have been implicated in muscle cell growth and differentiation. The expression patterns for these two genes were confirmed by qRT-PCR and their reduced expression over the developmental time course is in accord with postulated roles in the inhibition of muscle cell differentiation [22,23]. Both genes have been reported as early targets of MyoD expression in a mouse model of MyoD-induced differentiation [24]. In this model of myogenesis, *Fst1 *was down-regulated, whereas *Igfbp5 *was strongly up-regulated. This discrepancy with our results may indicate species-specific differences in *IGFBP5 *action in bovine muscle development, be due to the fact that our results are derived from an *in vivo*, not an *in vitro *model of myogenesis, or hint at the proposed dual role of this gene in the regulation of muscle cell development [25]. Our results lend further support to the notion that *FSTL1*, while its precise function is still unknown, has a crucial role in muscle development.

The *NEAT1*orthologue, a ncRNA species, is found at increasing levels during development of cattle muscle. *NEAT1 *was one of only three large abundant ncRNAs found enriched in the nucleus and highly conserved across mammalian species [18]. This, together with its association with SC35 splicing domains, suggests a fundamental function in mRNA metabolism for the *NEAT1 *transcript. The increasing representation of this ncRNA species in developing cattle muscle may be a reflection of the increasing number of nuclei in developing myofibres. Myonuclei accumulate in muscle cross-sections at an increasing rate up to about 180 days post conception [26]. The 6.8 fold higher representation of the *NEAT1 *orthologue transcript in the muscle of newborn cattle, compared with the muscle at d60, may therefore indicate a correspondingly higher proportion of nuclei per cell volume sampled. The observations of Ansay [27] on the DNA content of developing cattle muscle lend support to this view.

### Individual gene expression differences correlated with sire breed

While the microarray analysis detected a large number of genes that were significantly differentially expressed in at least one of the time points studied, only 2 of those genes showed a more than two-fold difference between the two sire breeds. We therefore examined the expression of myostatin (*GDF8*) itself by qRT-PCR. *GDF8 *RNA was expressed at significantly higher levels in the muscle of Piedmontese-sired fetuses at d 60 and d 135, in accordance with the findings of Forbes et al. [28] that the non-functional GDF8 molecule of the Piedmontese breed failed to repress the *GDF8 *promoter and therefore led to higher *GDF8 *transcript levels in the fetal muscle. Our observations therefore confirm that the *GDF8 *mutation did affect gene regulation in the fetal *longissimus *muscle as predicted.

One of the genes showing more than two-fold elevated expression in the muscle of newborn Wagyu-sired calves, *FABP5*, may indicate a higher level of adipogenic differentiation in the *longissimus *muscle of these animals, even at this early stage of their development. *FABP4 *showed a similar pattern of breed-specific differences, and the differential expression of both genes was confirmed by qRT-PCR (Tables 3 and 5). Fatty acid binding proteins are expressed at very high levels by adipocytes and are themselves, by interaction with peroxisome proliferator-activated receptors (PPAR) involved in mediating the effects of fatty acids on gene regulation [29]. Genetic variation in the bovine *FABP4 *gene has been found to be associated with intramuscular fat content and subcutaneous fat depth [30]. The possibility that FABP5 and FABP4 may be involved in defining the early onset of intramuscular fat deposition in the Wagyu breed remains to be explored. On the other hand, this finding may be an indication of relatively lower expression of *FABP4 *and *FABP5 *in Piedmontese-sired fetuses and therefore a consequence of the biochemical changes initiated by the myostatin mutation. In support of this latter possibility, a recent study of bovine fetuses carrying a myostatin mutation found a gene marker of adipocyte differentiation down-regulated in *semitendinosus *muscle at d 260 of gestation [11].

### Global gene expression differences correlated with sire breed

The microarray used in these experiments was based on adult muscle gene expression and is therefore less likely to detect expression differences in developmental regulatory genes involved with determining the double-muscled phenotype. However, the comprehensive view of developing muscle biochemistry that these experiments describes, points to subtle differences in timing of gene expression, as well as differences in the translational capacity of the Piedmontese muscle and an altered biochemical profile as the main consequences of the *GDF8 *mutation. Our study provides evidence for gene expression timing differences by the large number of ribosomal genes, which tend to be expressed at a relatively lower level in the *longissimus *muscle of Piedmontese-sired fetuses at the earlier time points and are then relatively more highly expressed at birth. This confirms the findings by Steelman et al. [31], who found genes involved in translation overrepresented among the up-regulated genes in 5-week-old myostatin null mice. A study of d 30- d 32 bovine embryos had identified a number of ribosomal protein genes as differentially expressed in embryos carrying the *GDF8 *mutation [32]. Cassar-Malek et al. [11], in a gene expression study in bovine *semitendinosus *muscle from d 260 fetuses carrying myostatin mutations also described higher levels of expression of ribosomal protein genes at this time point. Support for the view that timing differences characterize the development of *GDF8 *mutants comes from the work of Deveaux et al[33], which showed evidence for a delay in contractile differentiation of muscle from Belgian Blue fetuses, and Cagnazzo et al. [9], who showed that the fetuses from a more highly muscled breed of pigs showed a later peak of myogenesis-related gene expression during fetal development.

Our study confirms the findings by Cassar-Malek et al. [11] of decreased collagen gene expression in the muscle of late stage bovine fetuses carrying a myostatin mutation. This finding lends support to our interpretation of the temporal changes of collagen gene expression (above). As the *GDF8 *mutation results in larger numbers of muscle fibers that have larger cross-sectional areas on average, connective tissue fibroblasts (with collagen as the main part of their expression signature) should contribute relatively less to the overall gene expression profile.

*GDF8 *mutants are known to have a more glycolytic adult muscle phenotype than wild type animals [33] and this is clearly reflected in the gene expression comparisons between breeds at this time point. For example, the Piedmontese-sired fetuses show higher expression of genes that reflect fast, glycolytic fiber structural differentiation such as *ACTN3*, *MYH1 *and *MYH2*, and *MYBPC2*, coupled with higher expression of genes for glycolytic metabolic enzymes such as *ALDOA *and *LDHA*. These findings confirm the observations of Cassar-Malek et al[11] on bovine muscle and also of Steelman et al. [31], who documented a shift away from slow muscle fiber isoforms in myostatin null mice.

At birth, a suite of gene expression changes suggesting an increased ATP supply to the muscle of the Piedmontese-sired fetuses is observed. In fact, there is a coordinated up-regulation of genes encoding enzymes that determine mitochondrial activity at multiple steps in cellular respiration. These include key oxidative enzymes, in particular isoforms of cytochrome c oxidase (*COX5B*, *6C*, *7B*, *7C*), the terminal enzyme in the citric acid cycle. Cagnazzo et al[9] made a similar observation in their comparison of transcription profiles of developing muscles from two contrasting breeds of pigs. They found that a highly muscled breed of pigs (Pietrain) showed more active transcription of ATP metabolism-related genes during late fetal development.

This surge in ATP supply may reflect increased energetic demand in the immediate postnatal period in animals that experience very rapid postnatal growth, such as Piedmontese-sired calves or Pietrain piglets. Two major ATP consuming cellular processes are the maintenance of transmembrane ion gradients and protein turnover [34]. The demand for sarcolemmal ion gradients is unlikely to increase at this time. In fact, the expression of ATPase genes involved in this process (*ATP1A2*, *ATP2A1*, *ATP2A2*) is relatively lower in the muscle of Piedmontese-sired calves.

Therefore, taken together with the observation that a suite of ribosomal proteins is upregulated in Piedmontese-sired calves, we suggest that the extra ATP might be supporting more active ribosomal synthetic machinery. Consequently, the gene expression changes captured by this microarray appear to describe the energetic modifications necessary for the rapid muscular growth of newborn *GDF8 *mutants.

## Conclusion

This study describes the molecular events accompanying skeletal muscle differentiation in the bovine, namely a coordinate down-regulation of extracellular matrix-related gene expression at the same time as increasing gene expression levels of structural and metabolic constituents of muscle fibers. This study also highlights the developmental expression pattern of *FSTL1 *and *IGFBP5*, which have previously been implicated in myogenesis regulation, as well as describing the changing representation of a recently-described ncRNA (*NEAT1 *orthologue) in developing cattle muscle. A large number of breed-related gene expression differences were detected by contrasting the development of Wagyu-sired and Piedmontese-sired fetuses. While no breed-related differences in regulatory pathways were detected in this study, it documents the differential timing and magnitude of gene expression that result in the more glycolytic muscle fiber profile as well as the lower level of intramuscular fat observed in animals carrying myostatin mutations.

## Methods

### Animals

All animal experimentation complied with the Animal Ethics requirements of NSW Agriculture/GARAS. Hereford cows were artificially inseminated or mated to one of 5 different Wagyu sires or one of 6 different Piedmontese sires. All Piedmontese sires were homozygous for the *GDF8 *missense mutation in exon 3 and none of the Wagyu sires carried the mutation. 3 Fetuses from each sire breed were recovered by caesarean section at around 60, 130 and 195 of gestation. 3 newborn calves per sire breed were euthanased by lethal injection within 24 hours of birth. Details of the sampled fetuses and calves were recorded [see Additional file 4]. *Longissimus *muscle was dissected immediately after death of the fetuses or calves and tissue samples snap frozen in liquid nitrogen.

### Microarray methods

The process for RNA extraction and purification was performed as described in Lehnert et al. [21]. Anti-sense RNA (aRNA) amplification was performed using the MessageAmp aRNA Kit (Ambion, Austin, TX). Indirect labeling procedures and hybridizations were performed as described in Lehnert et al. [21] with the following modifications. The cDNA was generated using a modified amino C6 dT random primer [35] (Geneworks, Adelaide, SA) and the reverse transcriptase Superscript III (Invitrogen, Carlsbad, CA). The cDNA (pre-labeling) and the post-labeled cDNA were purified using the QIAquick PCR purification columns (Qiagen, Valencia, CA). The hybridization mixture used a reduced detergent concentration of 0.2%SDS.

We utilized a bovine cDNA microarray constructed from two cattle cDNA libraries; *longissimus *muscle and subcutaneous fat tissue derived from a 24 mo-old grass-fed Angus steer [21]. The array contained 9,600 bovine cDNA probes, printed in duplicate.

### Experimental design and data acquisition criteria

The weight of the individual fetuses [see Additional file 4], was analyzed by fitting an ANOVA model that contained the effects of breed, time, sex, and breed by time interaction. The ANOVA model accounted for 97.8% of the total variation in fetus weight and significant effects included time (P < 0.001), breed by time (P < 0.05) and breed (P < 0.10). The design of the microarray experiment therefore focused on the developmental aspect of the study, as well as exploring sire breed.

**Figure 3:**
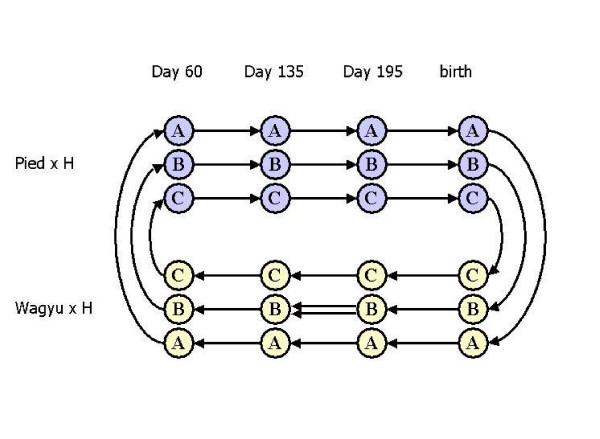
**Microarray experimental design**. Each microarray assay is symbolized by an arrow connecting the two *longissimus *muscle RNA samples that were hybridized to the array. The direction of the arrow indicates which sample was labeled with Cy3 (arrowhead) and Cy5 (origin).

Factors considered in designing the experiment included the availability of RNA for each sample of interest as well as the cost of the arrays themselves. Figure 3 illustrates the configuration layout for the experimental design that was developed with a total of three biological replicates, two breeds and four time points that were compared using 25 microarray hybridizations. The design was chosen to allow a focus on the developmental aspects of the study, but to also permit a breed comparison to be carried out. The entire set of expression data was deposited on the GEO database [14] and can be accessed using accession numbers GPL4196 and GPL4197.

We used the GenePix 4000A optical scanner (Molecular Devices, Sunnyvale, CA) and the GenePixPro 5.1 image analysis software (Molecular Devices) to quantify the gene expression level intensities, employing editing criteria detailed in [36]. Editing criteria were applied separately for the red (Cy5) and for the green (Cy3) intensity channels so that a different number of observations for each channel were obtained. These resulted in a total of 887,706 gene expression intensity readings (443,292 red and 444,414 green) that were background corrected and base-2 log transformed. The arithmetic mean and standard deviation (in brackets) for the red and green intensities were 11.93 (2.02) and 11.97 (1.95), respectively.

### Mixed-model equations and identification of differentially expressed genes

The following linear mixed-effect model was fitted to the data:

*Y*_*ijktmn *_= *μ *+ *C*_*ijk *_+ *G*_*m *_+ *AG*_*ijm *_+ *DG*_*km *_+ *TG*_*tm *_+ *ε*_*ijktmn*_

where *Y*_*ijktmn *_represents the *n*-th background-adjusted, normalized base-2 log-intensity from the *m*-th gene (probe) at the *t*-th treatment (fetal age and breed sample) from the *i*-th array, *j*-th printing block and *k*-th dye channel; *C *represents a comparison group fixed effect defined as those intensity measurements from the same array slide, printing block and dye channel; *G *represent the random gene (probe) effects with 8,845 levels; *AG*, *DG*, and *TG *are the random interaction effects of array × gene, dye × gene, and treatment × gene, respectively. Finally, ε is the random error term.

Variance components for random effects were estimated using restricted maximum likelihood. Differentially expressed (DE) genes were identified after processing the appropriate linear combination of the solutions (best linear unbiased predictions) of *TG via *model-based clustering, addressing the developmental as well as the breed comparison contrasts of interest [37,38].

A total of six contrasts were considered in the identification of DE genes. These included the two within-breed over-time contrasts and the four within-time across-breed contrasts. For each contrast, a two-component normal mixture model was fitted and posterior probabilities of belonging to the non-null component used to identify DE genes for an estimated experiment-wise false discovery rate of < 1% were computed as described by McLachlan et al. [39].

### Synthesis of complementary DNA (cDNA) for quantitative reverse transcription PCR (qRT-PCR) validation

TRIzol-extracted (Invitrogen) total RNA from *longissimus *muscle samples was further purified on RNeasy mini columns and treated on-column with DNase I (Qiagen) according to the manufacturer's instructions. Total RNA concentrations and relative purity were determined spectrophotometrically by measurement of UV absorbance at 260 and 280 nm (GeneQuant; GE Healthcare, Little Chalfont, UK). Reverse transcription of 1.5 μg of total RNA was performed with the Omniscript cDNA synthesis kit (Qiagen) using a mixture of oligonucleotides [100 μM OligodTVN (5'-TTTTTTTTTTTTTTVN-3' where V = A,C,G and N = A,C,G,T) and 1 μM 18Sprimer (5'-CACACGCTGAGCCAGTCAGT-3')]. The cDNA was diluted 1:10 in 10 mM Tris-HCl (pH8.0) for target gene measurements, whereas the 18S rRNA reference gene measurements were performed on a 1:200 dilution of cDNA.

Total RNA from d 60 fetal muscle samples and amplified RNA from the d 60 samples that were used to probe the DNA microarray were separately reverse transcribed. 1.5 μg of total or amplified RNA was reverse transcribed with the Superscript III first strand cDNA synthesis kit (Invitrogen). The cDNA synthesis used random hexamers and was performed at 37°C for 1 hr in the presence of RNase inhibitor (RNaseOUT, Invitrogen). The cDNA was diluted 1:10 in 10 mM Tris-HCl (pH8.0) and was used to measure the mRNA levels of *MATR3 *and *RPLP0*.

### qRT-PCR analyses

qRT-PCR to measure mRNA transcript levels was performed as described in Nattrass et al. [40], using the primer sequences listed in Table 6. qRT-PCR measurements were performed in triplicate on each cDNA sample (n = 24) on two separate occasions or once on the d 60 amplified and unamplifed samples (n = 12). Relative transcript quantitation was performed using standard curves generated for each gene from a 10-fold serial dilution of cDNA. Pooled cDNA from a subset of the *longissimus *muscle samples examined in this study was used to generate the standard curves. The standard curve dilutions, preparation and dispensing of the SYBR green I reagent and addition of the cDNA standards, reference cDNAs and cDNA samples were performed with a CAS1200 liquid handling robotics system (Corbett Life Science, Sydney, Australia). Target gene expression data from samples collected at day 60, day 135, day 195 and birth were determined by normalisizing against the measurements for 18SrRNA. Matrin 3 expression dataon the unamplified and amplified day 60 samples were determined by normalisizing against the measurements for RPLP0.

**Table 6:** Oligonucleotides used in quantitative reverse transcription PCR (qRT-PCR)

Gene	Symbol	sense	sequence	Genbank accession number
Myostatin (growth differentiation factor 8)	*GDF8*	FOR^1^	ACCTTCCCAGAACCAGGAGAA	AF019620
		REV	TCACAATCAAGCCCAAAATCTCT	
Fatty acid binding protein 4 (adipocyte)	*FABP4*	FOR	TGGAAACTTGTCTCCAGTGAAA	X89244
		REV	ACCCCCATTCAAACTGATGA	
Fatty acid binding protein 5 (psoriasis-associated)	*FABP5*	FOR	TGGGAGAGAAGTTTGAAGAGA	BT020981
		REV	TTCCTGATGTTGAACCAATGC	
Follistatin-like 1	*FSTL1*	FOR	TGCAGACCAGGAGAACAACA	BC114758
		REV	GGTTGAGGCACTTGAGGAAC	
Insulin-like growth factor binding protein 5	*IGFBP5*	FOR	GGTTTGCCTGAACGAAAAGA	S52657
		REV	CTTGGGCGAGTAGGTCTCC	
Matrin 3	*MATR3*	FOR (1)	GGAAAAAAGAACCTTCAGACA	CB434458
		FOR (2)	GACAAAGCTGTGAAAAAAGAT	
		REV	CCTCGATCTTGTCCACCTTT	
18S ribosomal RNA	*18SrRNA*		CGGTCGGCGTCCCCCAACTT	AY779625
			GCGTGCAGCCCCGGACATCTAA	
Ribosomal protein large, P0	*RPLP0*	FOR	CAACCCTGAAGTGCTTGACAT	NM001012682
		REV	GCAAGTGGGAAGGTGTAATCA	

FOR = forward primer; REV = reverse primer

### Statistical assessment of qRT-PCR gene expression data

Statistical analyses were performed using the Procedure GLM of SAS (SAS v9.1, SAS Institute Inc., Cary NC, USA) with an ANOVA model that included the expression of the genes (both target and reference genes), as measured by their threshold cycle (C_t_) of the PCR reaction, as dependent (response) variable and biological replicate (with two levels), breed (with two levels), age (with four levels), breed by age interaction (with eight levels) and residual as independent variables. Biological replicates were fitted as nested within breed by age interaction. Least square means of Ct for each gene and at each level of breed, age, and breed by age interaction were compared for differential expression. To ensure overall protection level, only probabilities associated with pre-planned comparisons were used.

## Authors' contributions

SAL participated in the study design, sample collections and data analysis and drafted the manuscript. AR participated in the microarray experimental design and carried out the statistical analyses. KAB participated in the microarray experimental design and analysis, and carried out RNA extractions and microarray experimentation. YHW participated in the study design and coordination. GSN carried out quantitative PCR validation. NJH helped to draft the manuscript. PLG was instrumental in developing the concepts for this study, and participated in the sample collections and helped to draft the manuscript. All authors read and approved the final manuscript.

## Supplementary Material

Additional file 1 Statistical thresholds for t-statistics and fold changes at two levels of significance and for down- and up-regulated genes. Statistical cut-off values for inclusion in differentially expressed gene lists Click here for file

Additional file 2 Genes showing differential gene expression in LM of bovine fetuses from different sire breeds in at least one time point. Full listing of all genes that showed differential expression between the two sire breeds. Click here for file

Additional file 3 Goodness of fit of the ANOVA model and significance of design effects on the expression of target and housekeeping genes as measured by the Ct of the qRT-PCR. Background detail to the analysis of qRT-PCR data. Click here for file

Additional file 4 Bovine fetuses sampled for microarray study. Listing of genetic background, weight, sex and accurate ages of the fetuses used in this study. Click here for file
