# Supplementary material for: Gene expression studies of developing bovine longissimus muscle from two different beef cattle breeds
Source: BMC Dev Biol. 2007 Aug 16;7:95. doi: 10.1186/1471-213X-7-95 (PMC2031903; doi:10.1186/1471-213X-7-95)
Supplement: Additional file 1 — Statistical thresholds for t-statistics and fold changes at two levels of significance and for down- and up-regulated genes. Statistical cut-off values for inclusion in differentially expressed gene lists [file 1471-213X-7-95-S1.doc]

Additional file 1: Statistical thresholds for t-statistics and gene expression ratios at two levels of significance and for down- and up-regulated genes^1^

| Contrast^2^ | Down-regulation | | | | Up-regulation | | | |
| --- | --- | --- | --- | --- | --- | --- | --- | --- |
|  | P < 0.05 | | P < 0.01 | | P < 0.05 | | P < 0.01 | |
|  | t-stat | ratio | t-stat | ratio | t-stat | ratio | t-stat | ratio |
| OTP | -0.45 | 0.73 | -0.54 | 0.69 | 0.39 | 1.31 | 0.44 | 1.35 |
| OTW | -0.55 | 0.68 | -0.62 | 0.65 | 0.33 | 1.26 | 0.41 | 1.33 |
| PW1 | -0.73 | 0.60 | -0.89 | 0.54 | 0.44 | 1.36 | 0.58 | 1.49 |
| PW2 | -0.36 | 0.78 | -0.44 | 0.74 | 0.44 | 1.36 | 0.52 | 1.43 |
| PW3 | -0.77 | 0.59 | -0.86 | 0.55 | 0.67 | 1.59 | 0.75 | 1.68 |
| PW4 | -0.40 | 0.76 | -0.51 | 0.70 | 0.27 | 1.21 | 0.38 | 1.30 |

^1^Only t-statistics values (with N = 8,845; for as many distinct probes on the microarray) were used to assess statistical significance. Significance was evaluated from the posterior probability of belonging to the cluster of extreme values in a model-based cluster analysis via mixtures of distributions (see Methods). Fold changes are back-solved from two to the power the value at the t-statistic.

^2^OTP: over-time in Piedmontese; OTW: over-time in Wagyu; PW1: Piedmontese versus Wagyu at 60 d gestation; PW2: Piedmontese versus Wagyu at 135 d gestation;

PW3: Piedmontese versus Wagyu at 195 d gestation; PW4: Piedmontese versus Wagyu at birth.
