# Supplementary material for: Gene expression studies of developing bovine longissimus muscle from two different beef cattle breeds
Source: BMC Dev Biol. 2007 Aug 16;7:95. doi: 10.1186/1471-213X-7-95 (PMC2031903; doi:10.1186/1471-213X-7-95)
Supplement: Additional file 2 — Genes showing differential gene expression in LM of bovine fetuses from different sire breeds in at least one time point. Full listing of all genes that showed differential expression between the two sire breeds. [file 1471-213X-7-95-S2.doc]

Additional file 2: Genes showing differential expression in LM of bovine fetuses from different sire breeds in at least one time point^1^

| Gene^2^ | GenBank  Accession^3^ | Number of array elements^4^ | Gene expression ratio^5^  P60d/W60d | Gene expression ratio^5^ P135 d/W135d | Gene expression ratio^5^ P195d/  W195d | Gene expression ratio^5^ Pbirth/ Wbirth |
| --- | --- | --- | --- | --- | --- | --- |
| actinin, alpha 2 (*ACTN2*) | CF614366 | 1 | 1.59 | 0.95 | 1.29 | 0.63** |
| actinin, alpha 3 (*ACTN3*) | CF614924 | 1 | 0.91 | 0.82 | 1.92** | 0.75* |
| aldehyde dehydrogenase 2 family (mitochondrial) (*ALDH2*) | CF614747 | 1 | 1.58** | 1.00 | 0.86 | 0.96 |
| Aldolase A, fructose-biphosphate (*ALDOA*) | CF615006 | 4 | 0.87 | 1.04 | 2.16** | 0.90 |
| ankyrin repeat domain 1 (cardiac muscle) (*ANKRD1*) | CF614403 | 2 | 0.87 | 1.13 | 1.70** | 0.96 |
| ATPase, Na+/K+ transporting, alpha 2 (+) polypeptide (*ATP1A2*) | CF614678 | 2 | 1.04 | 0.99 | 0.97 | 0.58** |
| ATPase, Ca++ transporting, cardiac muscle, fast twitch 1 (*ATP2A1*) | CF614616 | 1 | 0.72 | 0.87 | 1.11 | 0.54** |
| ATPase, Ca++ transporting, cardiac muscle, slow twitch 2 (*ATP2A2*) | CF614415 | 1 | 1.56** | 0.96 | 1.13 | 0.92 |
| ATP synthase, H+ transporting, mitochondrial F1 complex, alpha subunit, isoform 1, cardiac muscle (*ATP5A1*) | DW521775 | 1 | 1.55** | 1.12 | 1.10 | 0.61** |
| ATP synthase, H+ transporting, mitochondrial F1 complex, beta polypeptide (*ATP5B*) | CF615241 | 2 | 1.06 | 1.24 | 1.77** | 1.25* |
| ATP synthase, H+ transporting, mitochondrial F1 complex, epsilon subunit (*ATP5E*) | DW521469 | 3 | 0.88 | 0.90 | 0.85 | 1.75** |
| mitochondrially encoded ATP synthase 6 (*MT- ATP6*) | CO729228 | 4 | 0.94 | 1.10 | 0.80 | 1.31** |
| carbonic anhydrase III, muscle specific (*CA3*) | CF615449 | 16 | 1.10 | 1.02 | 1.01 | 0.51** |
| creatine kinase, muscle (*CKM*) | CF614897 | 7 | 0.92 | 1.03 | 1.69** | 0.84 |
| collagen, type I, alpha 1 (*COL1A1*) | CF613531 | 10 | 0.84 | 0.62** | 0.94 | 0.76* |
| collagen, type I, alpha 2 (*COL1A2*) | CF613956 | 6 | 1.36** | 0.67** | 1.02 | 0.53** |
| collagen, type III, alpha 1 (*COL3A1*) | CF613510 | 19 | 1.21 | 0.78* | 0.82 | 0.53** |
| collagen, type XII, alpha 1 (*COL12A1*) | CF614200 | 1 | 1.34 | 0.68** | 1.06 | 0.82 |
| collagen, type XV, alpha 1 (*COL15A1*) | DW521810 | 1 | 1.30 | 0.75* | 0.95 | 0.58** |
| cytochrome c oxidase subunit Vb (*COX5B*) | DW521806 | 1 | 1.02 | 0.95 | 0.74 | 1.69** |
| cytochrome c oxidase subunit VIc (*COX6C*) | DW521801 | 3 | 0.83 | 0.91 | 0.84 | 1.81** |
| cytochrome c oxidase subunit VIIb (*COX7B*) | CF614925 | 1 | 0.96 | 1.08 | 0.89 | 1.79** |
| cytochrome c oxidase subunit VIIc (*COX7C*) | DW521789 | 3 | 0.90 | 1.07 | 0.91 | 1.74** |
| COX17 homolog, cytochrome c oxidase assembly protein (yeast) (*COX17*) | DW521802 | 1 | 0.93 | 0.92 | 0.83 | 1.69** |
| fatty acid binding protein 4, adipocyte *(FABP4*) | CF614083 | 3 | 1.01 | 0.70** | 0.90 | 0.98 |
| fatty acid binding protein 5 (*FABP5*) | CF613827 | 1 | 1.29 | 0.93 | 1.16 | 0.44** |
| fibrillin 1 (*FBN1*) | DW521505 | 3 | 1.54** | 0.81 | 0.87 | 0.54** |
| four and a half LIM domains 1 (*FHL1*) | CF614427 | 11 | 1.54** | 1.03 | 1.27 | 0.62** |
| Fibronectin 1 (*FN1*) | DW521783 | 2 | 1.25 | 0.65** | 0.72 | 0.53** |
| glyceraldehyde-3-phosphate dehydrogenase (*GAPDH*) | CF615221 | 19 | 0.85 | 1.13 | 1.88** | 0.98 |
| heat shock 70kDa protein 1A (*HSPA1A*) | CF614728 | 2 | 0.97 | 0.98 | 0.79 | 0.59** |
| heat shock 70kDa protein 8 (*HSPA8*) | CF764011 | 6 | 1.20 | 1.02 | 0.93 | 0.51** |
| heat shock 90kDa protein 1, alpha (*HSPCA*) | DW521787 | 2 | 1.20 | 1.01 | 1.05 | 0.59** |
| heat shock 90kDa protein 1, beta (*HSPCB*) | DW521794 | 1 | 0.75 | 1.21 | 1.71** | 0.64** |
| Lactate dehydrogenase A (*LDHA*) | CF614486 | 1 | 1.33 | 1.05 | 1.87** | 1.10 |
| Matrin 3 (*MATR3*) | CB434458 | 1 | 2.35** | 1.15 | 0.79 | 0.64** |
| Myoglobin (*MB*) | CF614525 | 8 | 0.69 | 1.12 | 1.77** | 0.98 |
| mitochondrially encoded NADH dehydrogenase 1 (*MT-ND1*) | CF613825 | 1 | 1.08 | 0.73** | 0.54** | 1.33** |
| mitochondrially encoded NADH dehydrogenase 2 (*MTND2*) | CF613937 | 1 | 1.09 | 1.09 | 0.61 | 1.39** |
| mitochondrially encoded NADH dehydrogenase 3 (*MTND3*) | DW521776 | 2 | 0.96 | 1.26 | 0.55** | 1.86** |
| myosin binding protein C, slow type (*MYBPC1*) | CF615188 | 3 | 1.51** | 0.92 | 0.93 | 0.66** |
| myosin, heavy polypeptide 1, skeletal muscle, adult (*MYH1*) | CF614905 | 1 | 0.84 | 0.82 | 1.89** | 0.77 |
| myosin, heavy polypeptide 2, skeletal muscle, adult (*MYH2*) | CF615247 | 1 | 1.32 | 0.91 | 1.58 | 0.61** |
| Myozenin 1 (*MYOZ1*) | CF614894 | 2 | 0.80 | 1.00 | 1.52 | 1.37** |
| NADH dehydrogenase (ubiquinone) 1 beta subcomplex, 3, 12kDa (*NDUFB3*) | CF615054 | 2 | 0.88 | 0.90 | 0.81 | 1.79** |
| Nebulin (*NEB*) | CF614405 | 3 | 1.53** | 0.99 | 1.04 | 0.67** |
| ribosomal protein L8 (*RPL8*) | DW521784 | 1 | 0.58* | 0.91 | 1.08 | 1.34** |
| ribosomal protein L13a (*RPL13a*) | CF613651 | 5 | 0.64 | 0.92 | 0.94 | 1.26* |
| ribosomal protein L17 (*RPL17*) | CF613790 | 1 | 0.60* | 0.80 | 0.90 | 1.45** |
| ribosomal protein L18 (*RPL18*) | CF613665 | 1 | 0.75 | 1.07 | 1.31 | 1.67** |
| ribosomal protein L18a (*RPL18A*) | CF614784 | 1 | 0.81 | 1.12 | 1.03 | 1.68** |
| ribosomal protein L19 (*RPL19*) | DW521809 | 1 | 0.91 | 0.97 | 1.02 | 1.84** |
| ribosomal protein L21 (*RPL21*) | CF613521 | 1 | 0.66 | 0.85 | 0.88 | 1.47** |
| ribosomal protein L22 (*RPL22*) | CF614058 | 1 | 0.81 | 0.92 | 0.97 | 1.87** |
| ribosomal protein L30 (*RPL30*) | DW521790 | 3 | 0.92 | 0.96 | 0.95 | 1.70** |
| ribosomal protein L31 (*RPL31*) | DW521637 | 2 | 0.90 | 0.86 | 0.91 | 1.68** |
| ribosomal protein L34 (*RPL34*) | DW521781 | 2 | 0.69 | 1.10 | 1.11 | 1.71** |
| ribosomal protein L35a (*RPL35A*) | DW521786 | 1 | 0.80 | 0.88 | 0.87 | 1.66** |
| ribosomal protein L36a (*RPL36A*) | CF613781 | 1 | 0.80 | 1.05 | 1.14 | 1.75** |
| ribosomal protein L37a (*RPL37A*) | CF613632 | 6 | 0.70 | 0.94 | 0.97 | 1.63** |
| ribosomal protein L41(*RPL41*) | CF613750 | 2 | 0.88 | 0.96 | 0.94 | 1.70** |
| ribosomal protein S7 (*RPS7*) | CF614059 | 1 | 0.99 | 0.67** | 1.11 | 0.94 |
| ribosomal protein S8 (*RPS8*) | DW521811 | 1 | 0.71 | 0.66** | 0.71 | 1.08 |
| ribosomal protein S12 (*RPS12*) | DW521788 | 4 | 0.81 | 0.93 | 0.97 | 1.71** |
| ribosomal protein S15a (*RPS15A*) | DW521791 | 1 | 0.75 | 0.99 | 0.97 | 1.67** |
| ribosomal protein S25 (*RPS25*) | DW521792 | 4 | 0.79 | 0.96 | 1.11 | 1.72** |
| ribosomal protein S26 (*RPS26*) | DW521796 | 1 | 0.71 | 1.08 | 1.13 | 1.67** |
| ribosomal protein S28 (*RPS28*) | DW521805 | 1 | 0.86 | 0.93 | 0.93 | 1.78** |
| reticulon 4 (*RTN4*) | CF615173 | 8 | 1.25 | 0.99 | 1.12 | 0.55** |
| stearoyl-CoA desaturase (delta-9-desaturase) (*SCD*) | CF613504 | 18 | 1.60** | 0.75* | 1.04 | 0.59** |
| secreted protein, acidic, cysteine rich (osteonectin) (*SPARC*) | CF614039 | 1 | 1.27 | 0.63** | 0.76 | 0.77 |
| testis enhanced gene transcript (BAX inhibitor 1) (*TEGT*) | CO729239 | 3 | 1.19 | 0.88 | 1.39 | 0.58** |
| TIMP metallopeptidase inhibitor 3 (*TIMP3*) | DW521779 | 1 | 1.27 | 0.77* | 1.22 | 0.55** |
| thymosin, beta 10 (*TMSB10*) | DW521778 | 2 | 0.69 | 0.82 | 0.85 | 1.31** |
| thioredoxin interacting protein (*TXNIP*) | DW521795 | 2 | 1.58 | 0.74** | 0.88 | 0.86 |
| Vimentin (*VIM*) | DW521780 | 1 | 0.93 | 0.60** | 0.81 | 0.97 |

^1^ * indicates a statistically significant gene expression difference at P < 0.05 level; ** indicates a statistically significant gene expression difference at P < 0.01 level.

^2^Sequence annotation was carried out using approved gene symbols (human genome nomenclature) *via* the IBISS4 database [15]. BLAST [17] scores > 200 and e-values < 1E-50 were accepted for annotation purposes.

^3^In cases where more than 1 microarray element was used to calculate the expression data, the accession number of 1 representative element is shown.

^4^Number of microarray elements representing the same gene, that were used to calculate the expression data.

^5^To obtain the ratios shown, averaged absolute microarray signal intensity values for P (Piedmontese x Hereford) fetuses were divided by the signal intensities measured for W (Wagyu x Hereford) fetuses at d 60, d 135, d 195 and birth.
