# Supplementary material for: Gene expression studies of developing bovine longissimus muscle from two different beef cattle breeds
Source: BMC Dev Biol. 2007 Aug 16;7:95. doi: 10.1186/1471-213X-7-95 (PMC2031903; doi:10.1186/1471-213X-7-95)
Supplement: Additional file 3 — Goodness of fit of the ANOVA model and significance of design effects on the expression of target and housekeeping genes as measured by the Ct of the qRT-PCR. Background detail to the analysis of qRT-PCR data. [file 1471-213X-7-95-S3.doc]

Additional file 3: Goodness of fit of the ANOVA model and significance of design effects on the expression of target and housekeeping genes as measured by the Ct of the qRT-PCR

| Gene | Replicate | Breed | Age | Breed*Age | R^2^, % |
| --- | --- | --- | --- | --- | --- |
| IGFBP5 | N.S. | * | *** | *** | 87.7 |
| FSTL1 | N.S. | *** | *** | *** | 94.1 |
| GDF8 | N.S. | *** | *** | N.S. | 88.9 |
| FABP4 | N.S. | *** | *** | *** | 80.7 |
| FABP5 | *** | ** | *** | *** | 64.5 |
| 18S RNA | N.S. | N.S. | ** | *** | 38.3 |

Biological replicated fitted nested within Breed by Age (Breed*Age) interaction.

^*^Significance at P < 0.05; ^**^Significance at P < 0.01; ***Significance at P < 0.001; N.S. = non-significance (P > 0.05).
