# Supplementary material for: Gene expression studies of developing bovine longissimus muscle from two different beef cattle breeds
Source: BMC Dev Biol. 2007 Aug 16;7:95. doi: 10.1186/1471-213X-7-95 (PMC2031903; doi:10.1186/1471-213X-7-95)
Supplement: Additional file 4 — Bovine fetuses sampled for microarray study. Listing of genetic background, weight, sex and accurate ages of the fetuses used in this study. [file 1471-213X-7-95-S4.doc]

Additional File 4: Bovine fetuses sampled for microarray study

| **Fetus** ^1^ | **sex** | **Cow ID** | **Sire ID** ^2^ | **D p.c.**^3^ | **Weight** |
| --- | --- | --- | --- | --- | --- |
| D 60 WxH 1 | n/d | 5699906 | W006 | 60 d | 12.31g |
| D 60 WxH 2 | Female | 5699911 | W006 | 60 d | 13.32g |
| D 60 WxH 3 | Female | 5699597 | W006 | 61 d | 14.03g |
| D 60 PxH 1 | n/d | 5699663 | DMR7 | 60 d | 16.46g |
| D 60 PxH 2 | Male | 5699904 | DMR7 | 62 d | 15.96g |
| D 60 PxH 3 | Female | 5699546 | DMR7 | 61 d | 12.91g |
| D 135 WxH 1 | female | 5595625 | W006 | 133 d | 1.26 kg |
| D 135 WxH 2 | female | 5699935 | W006 | 138 d | 1.44 kg |
| D 135 WxH 3 | male | 2192780 | W006 | 138 d | 1.94 kg |
| D 135 PxH 1 | male | 5699672 | DMP45 | 132 d | 2.50 kg |
| D 135 PxH 2 | female | 5699921 | DMQ2 | 132 d | 1.82 kg |
| D 135 PxH 3 | female | 5591012 | DMQ2 | 128 d | 1.18 kg |
| D 195 WxH 1 | male | 5393024 | W6 | 193 d | 8.96 kg |
| D 195 WxH 2 | male | 1392067 | W5 | 195 d | 8.54 kg |
| D 195 WxH 3 | female | 5699600 | W3 | 194 d | 8.26 kg |
| D 195 PxH 1 | male | 1390007 | DM3 | 195 d | 9.02 kg |
| D 195 PxH 2 | female^4^ | 5393041 | DM3 | 194 d | 7.74 kg |
| D 195 PxH 3 | male^4^ | 5393041 | DM3 | 194 d | 7.56 kg |
| birth WxH 1 | female | 5699682 | W3 | 279 d | 32.0 kg |
| birth WxH 2 | female | 1390004 | W5 | 279 d | 30.0 kg |
| birth WxH 3 | female | 5699926 | W1 | 277 d | 32.5 kg |
| birth PxH 1 | female | 5699896 | DM5 | 287 d | 33.0 kg |
| birth PxH 2 | female | 5699881 | DM6 | 279 d | 48.0 kg |
| birth PxH 3 | female | 5699587 | DMP45 | 280 d | 42.0 kg |

^1^ P = Piedmontese; H = Hereford; W = Wagyu; ^2^ W= Wagyu sire; DM=Piedmontese sire (homozygous for *GDF8* mutation); ^3^ d *post conception*; ^4^twins
